# Influence of crowd size on home advantage in the Japanese football league

**DOI:** 10.3389/fspor.2022.927774

**Published:** 2022-09-09

**Authors:** Ryota Nomura

**Affiliations:** Faculty of Human Sciences, Waseda University, Tokyo, Japan

**Keywords:** home advantage, crowd size, football, structural equation modeling, natural experiment

## Abstract

This study aimed to investigate the influence of crowd size on home advantage (HA). Data of the 2019 and 2020 seasons of the J1 League (Japan Professional Football's First Division League) were analyzed. Matches during the 2019 season were played under regular conditions, while there was low stadium occupancy during the 2020 season to prevent the spread of COVID-19. Regarding average points won, HA disappeared. By using multiple group structural equation modeling, it was examined the routes of influence *via* which a reduction in crowd size influenced HA. The results indicated that the influence from the crowd size to the referee's decisions disappeared during the 2020 season. However, the factors including the referee's decisions have lower effects on the outcome factor. Hence, no dominant route was detected in the present study.

## Introduction

Home advantage (HA) is a phenomenon wherein a home team has an advantage over an away team. Schwartz and Barsky (1977)—often cited as authors of early research on HA—defined that HA is a phenomenon whereby home victories exceed 50% of all matches won when the schedule of matches held at home and away is balanced. The researchers noted that HA occurred in several professional sporting events at the time. The results of professional sports at the time indicated marked HA for indoor sports such as basketball and hockey but poor HA for the outdoor sports of football and baseball (Schwartz and Barsky, 1977; Losak and Sabel, 2021). However, in recent years, values modified by Pollard's method have been used to determine HA. These focus on the degree of deviation from the winning percentage of 50% (Matos et al., 2020). Researchers have highlighted that HA is consistently seen in regular season football league matches (Almeida and Leite, 2021; Leitner and Richlan, 2021).

Courneya and Carron (1992) have cited four major game location factors thought to affect the degree of HA: (1) learning/familiarity factors; (2) rule factors; (3) travel factors; (4) crowd factors. The learning/familiarity factors pertain to the home team's familiarity with the characteristics of the venue where a match is to be played, which positively affects their score. The travel factors concern players' performances and the effect of the following: (a) mental and physical fatigue resulting from the visiting team's travel to the next venue and the difference in the environment of hotels from everyday living; (b) changes in players' condition due to differences in climate, such as temperature and humidity, and differences in food culture on game results. However, some researchers observed that these influences have faded in recent years with the development of better means of transportation and improvements in hotel environments (Courneya and Chelladurai, 1991; Pace and Carron, 1992). Coincidentally, on 24 June 2021, the Union of European Football Associations (UEFA) announced the abolition of the away goals rule, which was applied to determine the winner of a two-legged knockout tie in cases where the two teams had scored the same number of goals on aggregate over the two matches. In such cases, the team which had scored the higher number of goals away from home was considered the winner of the tie and qualified for the next round of the competition. This rule had been in use since 1965 in UEFA Champions League competitions and had been put in place to correct for HA. The UEFA cited improvements in conditions since the rule had been adopted, a reduction in the gap in winning percentages, and the reduction in the average goals per match at home/away matches as reasons for abolishing the rule. The rule factor refers to the differences in the rules between the home and away teams that created an HA. However, the rule factor is said to have the weakest influence of the four factors, and the types of sport on which it shows an influence are limited (Courneya and Carron, 1992).

Of the four factors pointed out by Courneya and Carron (1992), crowd factors appear to have an especially strong influence on HA. Crowd factors relate to crowd density affecting the mental state of the home team, causing changes in the players' actions/behaviors and performances and bringing about HA consequently (Agnew and Carron, 1994). Other than exerting a direct influence on the players, crowd factors are perceived to influence the referees' decisions (Leitner and Richlan, 2021; Wunderlich et al., 2021). In both cases, the anticipated processes are the crowd's behaviors of cheering and supporting the home team and/or booing the away team, which creates an advantageous environment for the home team and the contrary for the away team.

Previous studies (e.g., Almeida and Leite, 2021; McCarrick et al., 2021) suggest that stadium-packed crowds influence HA and predicted that the effects of crowd factors would disappear in games held without any spectators. After 2020, the worldwide spread of COVID-19 created an opportunity to verify this prediction. Matches became a large-scale social experiment as they were held in the absence of spectators due to the restriction of crowds in stadiums. This created an opportunity to investigate the mechanism by which HA occurs. To verify the influence of matches without any spectators on HA, researchers have, since 2020, been actively conducting studies to compare the results of matches played in the leagues of various countries between the 2018–2019 season and the 2019–2020 season. The results, supported by most studies, indicate that matches played without spectators caused HA to disappear (For systematic review, see Leitner et al., 2022). For example, McCarrick et al. (2021) analyzed data from 15 leagues in 11 countries and reported that HA could no longer be seen in matches with no spectators. Other researchers have also reported that HA was no longer evident in many cases (Almeida and Leite, 2021, Germany, Spain, England, Portugal; Leitner and Richlan, 2021, Spain, England, Germany, Italy, Russia, Turkey, Austria, and the Czech Republic; Hill and Van Yperen, 2021, Spain, Italy, and England). However, some studies claimed that in certain cases, HA had been maintained in matches without any spectators (Almeida and Leite, 2021, Spain) and, conversely, reported that an HA had occurred (Tilp and Thaller, 2020, Germany). Overall, most studies reported the disappearance of HA.

These results suggest that HA is a function of crowd size. In addition to the previously described reports on the top leagues of various countries, it has been indicated that HA has not occurred in amateur leagues where crowd sizes tend to be small (Fischer and Haucap, 2021; Wunderlich et al., 2021), suggesting that HA is facilitated by crowd size. Major hypotheses put forward in past research can be broadly summarized as follows. First, spectators cheering for the home team increases the players' attacking opportunities during play, which contributes to capturing victory (McCarrick et al., 2021). Second, for the away team, conversely, having numerous spectators who cheer for their opponents and getting booed occasionally cause a reduction in their performance, which leads to their defeat (Greer, 1983). Third, referees face the risk of being booed for decisions that go against the home team because of the numerous spectators who support them (Nevill et al., 2002). This may exert a psychological influence on the referees, such as pressure, making them more reluctant to make decisions disadvantageous to the home team, such as handing out yellow and red cards (Leitner and Richlan, 2021; Wunderlich et al., 2021).

**Figure 1:**
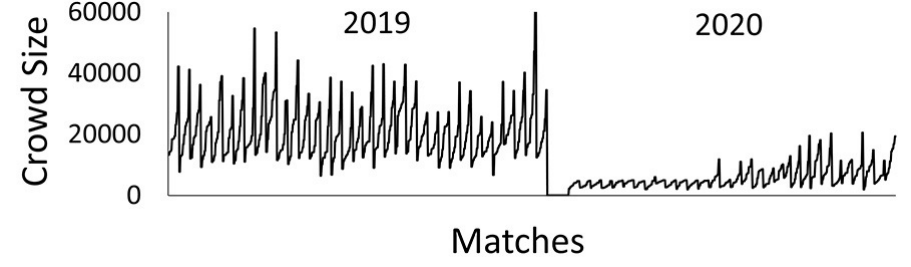
The fluctuations of crowd size during 2019 and 2020 seasons. In the round 2 and 3 of 2020 season, there were no spectators in all matches.

In this study, I have used data from the Japan Professional Football's First Division League (hereafter J1 League) to investigate these hypotheses. I used a point-based system to examine whether HA disappeared in 2020 when the number of spectators was restricted. Here, I employed points, rather than percentages of wins and their variations modified using the Pollard method (Matos et al., 2020). This is because points are indices for determining ranking within a league: they affect whether a team remains within the league or is promoted. Next, I built a structural equation model that hypothesized that the crowd size influences team activity (running distance, number of sprints), team performances (number of goal shots, corner kicks), and the referee's decisions (number of warnings issued and send-offs). Data of the 2019 season, wherein the league operated as usual, were compared with those of the 2020 season, wherein matches were played with smaller crowds, on account of COVID-19. The J1 League eased the restrictions placed on the number of spectators during the 2020 season in phases. Thus, the crowds gradually increased in size in response to the infection countermeasures implemented at each time point (Figure 1). A statistically significant influence must be found vis-à-vis various types of explanatory variables unique to each game to determine whether crowd size is a dominant variable. Therefore, I statistically verified (a) the primary route by which the crowd size influences points through player performances and (b) the secondary route by which the crowd size influences points through referee decisions both by season (2019 or 2020) and by game location (home or away).

## Methods

### Targeted matches

In this study, I used the results of matches played (305 and 298) during both the 2019 and 2020 seasons of the J1 League as my targets of analysis, the results of which are reflected in rounds 1–34. The matches played during the 2019 season were treated as the data for regular matches, and those played during the 2020 season were treated as the data for matches in which a limit was placed on crowd size. Matches played from rounds 2 and 3 in 2020, however, were played without any supporters. During the 2019 and 2020 seasons, the J1 League had 18 teams. Each time the season changes, the J1 League's bottom two teams are replaced by the J2 League's top two teams. Considering the impact of COVID-19, the League matches for the 2020 season took the form of “not demoting any teams but promoting teams” to secure fairness.

### Variables

I used the following variables of the home and away teams and compared them: number of points, goals, goal shots made, corner kicks, running distance, number of sprints, warnings, and send-offs. All the data were based on the database published by the J1 League. The definitions of variables are shown in Table 1.

**Table 1:** Definitions of variables.

*Crowd size*
The number of attendances that officially published in the database of the J1 League.
*Team activity*
- Running distance
The value that indicates the distance that a player in a team traveled during a game. The distance run by the 11 starting players, plus that of any substituted players, is totalled.
- Number of sprints
The value that indicates the number of moves that a player in a team makes for more than a set amount of time during a game. According to the J League's regulations, a sprint is recorded if a player continues to run at a speed of more than 24 km/hr for more than a second. As with running distance, the distance run as sprints by the 11 starting players, plus that of the players who entered the game midway, is totaled.
*Team performance*
- Number of goal shots
The number of attempts to score a goal. Other than plays clearly aimed at scoring a goal, plays that depart from what the player had intended, such as crosses and other balls threatening to score a goal, were also recorded as the number of goal shots made.
- Number of corner kicks
The number of set plays made when an opponent sends the ball from their own goal line. The game is resumed after the ball is kicked in from the corner of the field, located to the right and left of the goal, called the corner arc. A corner kick is unique in that it can be aimed directly at the goal.
*Referee's decisions*
- Number of warnings issued
The number of times a yellow card was presented during a game. A yellow card is presented in response to dangerous fouls or fair-play-violation. If it is presented twice during a game, the player is sent off.
- Number of send-offs
The number of times a player is ordered off the pitch on being presented with a yellow card twice or having a red card issued against them. A red card is issued when acts more dangerous or violent than those that warrant a yellow card take place.
*Outcome*
- Points
A numerical value that determines a team's ranking within the League, with three points for a winning game, one point for a draw, and zero for a losing game. The interval between points is made uneven to prevent the teams from gaming the system by aiming for draws rather than wins and motivate teams to engage in even more offensive play (Aylott and Aylott, 2007).
- Goals
The number of times a player puts the ball in the opponent team's goal.

### Analysis policies

First, a two-way analysis of variance (ANOVA) was performed to verify HA in the J1 League. It used seasons (2019 or 2020) × game locations (home or away) as the independent variables and points as the dependent variable. Second, a two-way ANOVA was performed to verify the independent effects of each variable. For this analysis, I used seasons (2019 or 2020) × game locations (home or away) as the independent variables and each variable of performance and referee judgement (points, goals, goal shots made, corner kicks, running distance, number of sprints, warnings, and send-offs) as the dependent variables. Finally, a SEM (structural equation modeling) was conducted to test whether crowd size predicted these variables and whether these variables, in turn, predicted the outcome variables.

**Figure 2:**
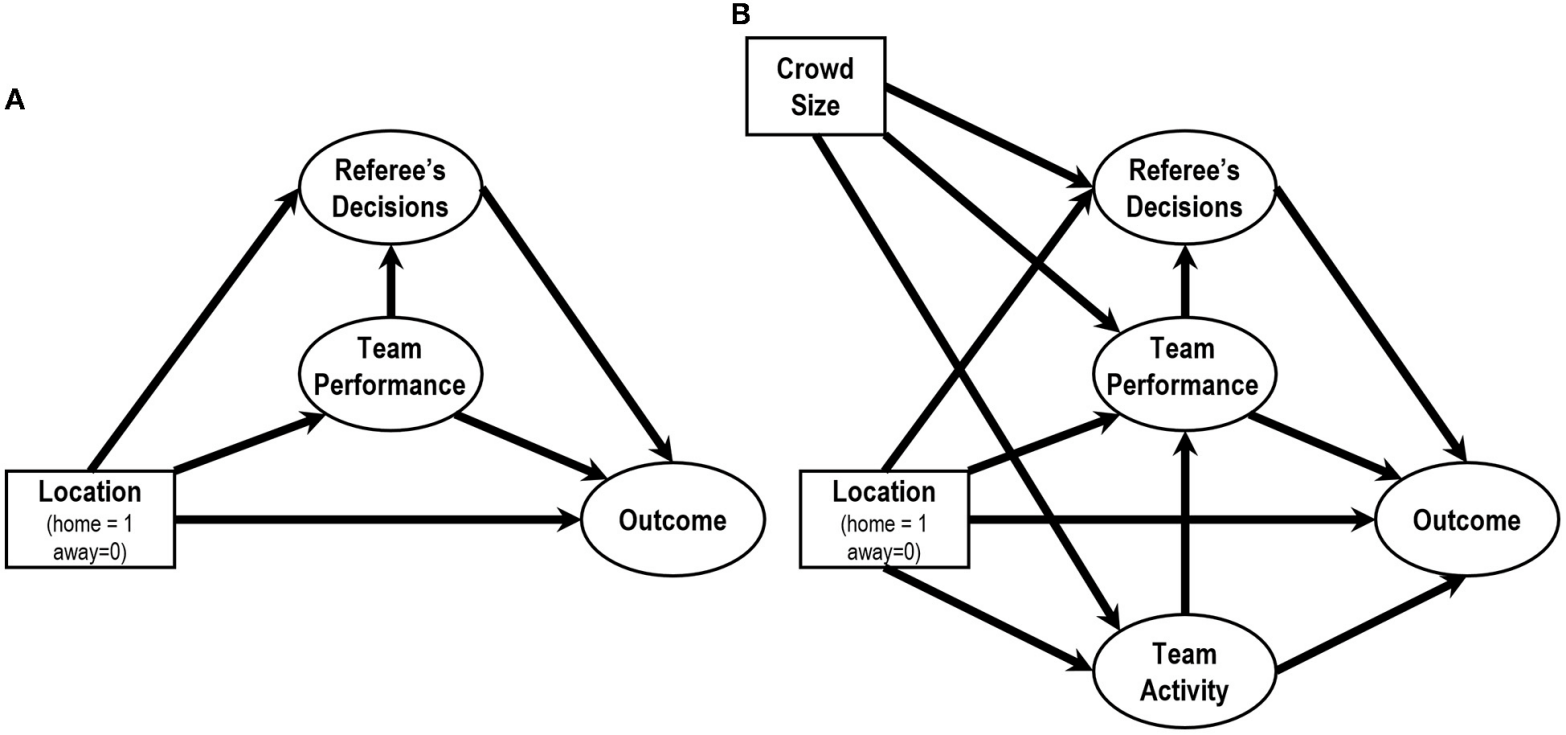
**(A)** The basic structure of home advantage mediated (HAM) model proposed by Bilalić et al. (2021) and **(B)** the model proposed in the present paper.

Regarding the process of HA, Bilalić et al. (2021) proposed home advantage mediated (HAM) model to represent the relationships among variables. In the first step of the model, HA predicts the factor of team performance (corner kicks, shots, and shots on target) and the factor referee's decisions (fouls, yellow cards, and red cards). In the second step, these factors predict the outcome factors (points and goals). The process would be applicable to data obtained from J1 League as well because the basic structure of HA would be consistent among football leagues. Thus, I adopt a model considering these basic relationships (Figure 2A), although the observed variables were not the identical to the original HAM model. Moreover, I then additionally consider a factor of team activity (running distance and the number of sprint) as a first step factor. This is because that the team performance factor would be determined by the observable variables of team activity. I therefore set a path from the factor team activity to the factor team performance.

Then, I originally add the observed variable crowd size as a variable that predict the above first step factors, i.e., the factors of team activity, team performance, and referee's decisions. This depends on the main assumption of this paper; The crowd size influences each player's and referee's behaviors, resulting outcomes such as points scored and goals. Finally, I construct a proposed model as shown in Figure 2B. In this model, I did not include the interaction between the game locations and the crowd size. In other words, these variables are treated as being independent of each other. To compare the results of 2019 and 2020 seasons directly, a multiple group SEM was applied to the dataset simultaneously. For these analyses, I used SPSS Statistics ver. 27 (IBM Corporation) and SPSS Amos ver. 27 (IBM Corporation).

## Results

### Descriptive statistics and summary of crowd size

Table 2 demonstrate the descriptive statistics of predictive variables and the outcome variables. Regarding the crowd size, the minimum value of the size was zero in round 2 and 3 in the 2020 season under the restriction due to the COVID-19. The maximal value 34,521 of 2020 was recorded in round 1, thus the number of spectators was not restricted within this period. The maximal value including both periods was 63,854 recorded at the match between Yokohama F. Marinos and F. C. Tokyo at the round 34 in the 2019 season. Figure 1 show the crowd size at each time points.

**Table 2:** Descriptive statistics of variables.

	**2019**					**2020**					**Full**				
	***Min*.**	***Mean***	***Med*.**	***Max***	***SD***	***Min*.**	***Mean***	***Med*.**	***Max***	***SD***	***Min*.**	***Mean***	***Med*.**	***Max***	***SD***
Crowd size	6,491	20,755	4,698	63,854	9,007	0	5,840	18,390	34,521	4,456	0	13,384	11,744	63,854	10318
Point	0	1.38	1.00	3	1.332	0	1.39	1.00	3	1.337	0	1.39	1.00	3	1.334
Goals	0	1.30	1.00	8	1.2	0	1.41	1.00	6	1.23	0	1.35	1.00	8	1.215
Running distance	95.94	112.14	114.22	127.8	4.725	99.84	114.5	111.78	131.4	5.129	95.94	113.3	113.01	131.4	5.064
Number of sprints	19	160.57	164.50	245	28.5	86	164.4	159.00	261	25.59	19	162.47	161.00	261	27.16
Number of GS	1	10.19	10.00	23	4.102	1	10.62	10.00	33	4.397	1	10.4	10.00	33	4.254
Number of CK	0	4.89	5.00	15	2.502	0	4.89	5.00	17	2.768	0	4.89	5.00	17	2.636
Number of WI	0	1.08	1.00	6	1.034	0	1.08	1.00	6	1.13	0	1.08	1.00	6	1.082
Number of send-offs	0	0.04	0.00	3	0.232	0	0.04	0.00	2	0.209	0	0.04	0.00	3	0.221

GS, goal shots; CK, coner kicks; FK, free kicks; WI, warnings issued.

### Examination of HA (a two-way ANOVA of points scored)

**Figure 3:**
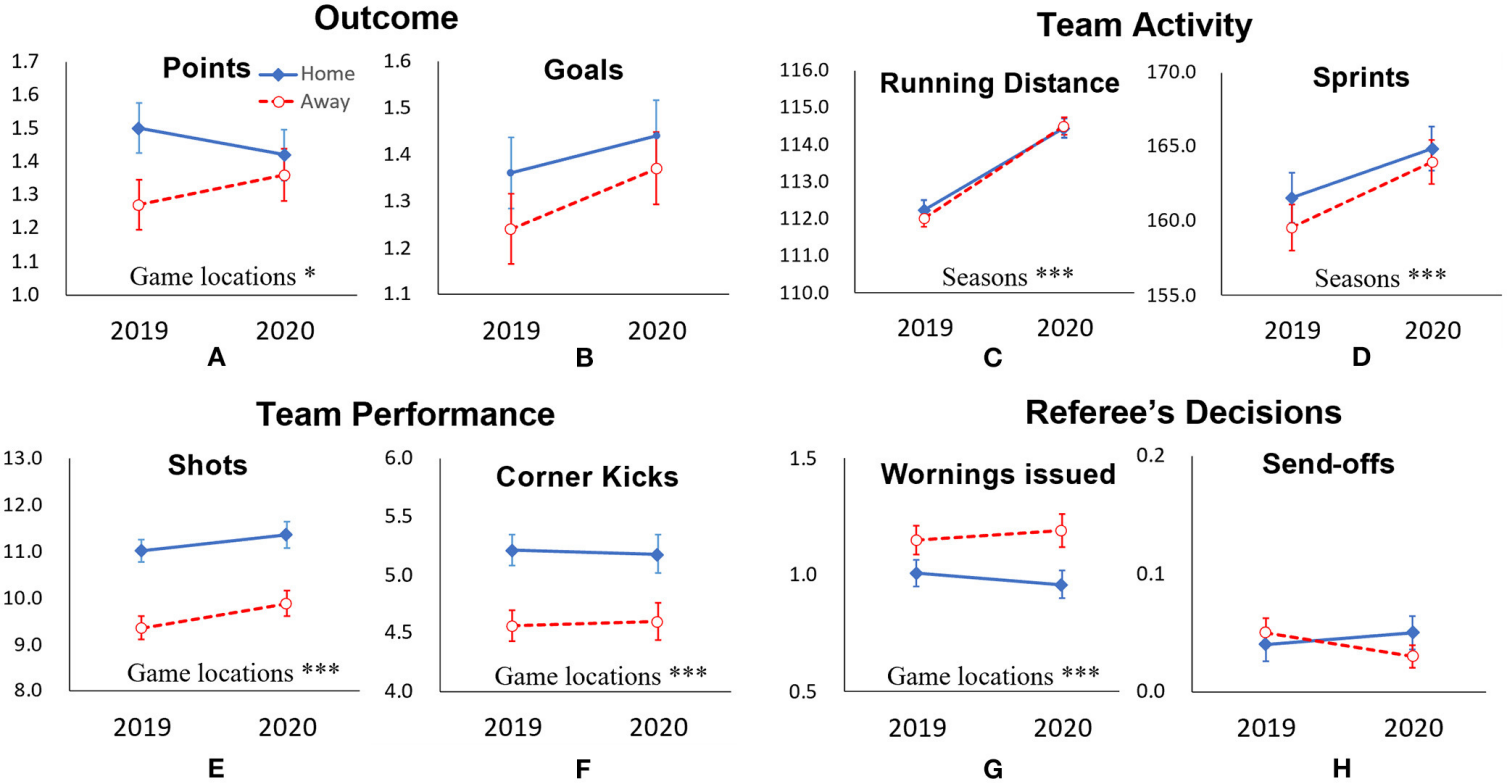
Descriptive statistics for seasons × game locations. Outcome (upper left panel): **(A)** average number of points scored per game and **(B)** average number of goals; Team Activity (upper right panel): **(C)** average number of the running distance and **(D)** average number of sprints; Team Performance (lower left panel): **(E)** average number of shots and **(F)** average number of corner kicks; and Referee's Decisions (lower right panel): **(G)** average number of warnings issued and **(H)** average number of send-offs. The independent variables described in insets represent that the main effect of the variable was significant. Error bars indicate ±1.0 standard error of the mean. *p < 0.05; ***p < 0.01.

A two-way ANOVA was performed using seasons (2019 or 2020) × game locations (home or away) as the independent variables and points scored as the dependent variable. As shown in Figure 3A, a significant main effect of game location was found in terms of points scored (*F*_(1,1202)_ = 3.56*, p* < 0.05). As a result of a simple main effects test, a significant difference was seen only in 2019 (*p* < 0.05). Conversely, differences in points scored according to game location seen in 2019 were no longer observed in 2020. This suggests the disappearance of HA in 2020.

### Two-factor ANOVA of variables

A two-way ANOVA was performed using seasons (2019 or 2020) × game locations (home or away) as the independent variables and the number of goal shots, corner kicks, running distance, number of sprints, warnings issued, and send-offs as the dependent variables. Figures 3B–H show the results of the analyses. A significant difference was observed consistently, with the values of the number of goal shots and corner kicks being higher at home (Home > Away in both 2019 and 2020). Conversely, the number of warnings issued was greater in away matches (Away > Home in both 2019 and 2020). A significant difference was also observed with the running distance and the number of sprints being higher in the 2020 season at both home and away matches. Other variables were not significant in terms of either main effects or interactions.

### A process model analysis

As the result of a multiple group SEM assuming the same structure between 2019 and 2020, the goodness of fit indices exhibited that the model was acceptable (*CMIN/df* = 2.82, *GFI* = 0.974, *AGFI* = 0.950, *CFI* = 0.931, and *RMSEA* = 0.039).

**Figure 4:**
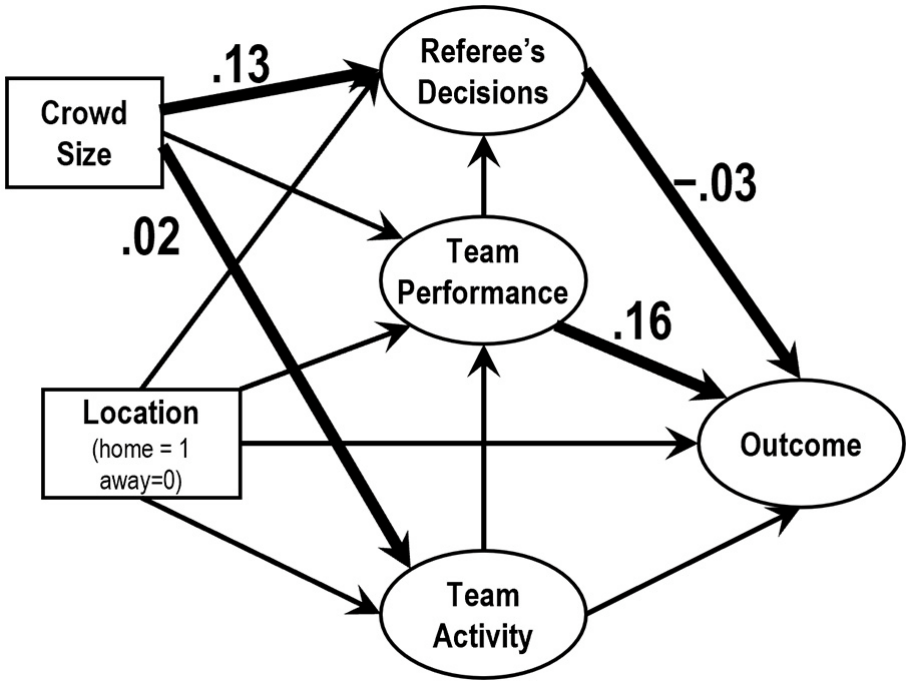
The results of SEM of data observed during 2019 season. The numbers show the statistically significant (*p* < 0.05) normalized coefficients. Non-significant coefficients were omitted.

Figure 4 depicts the SEM using matches played in 2019. The crowd size significantly influenced the factor referee's decision (0.13); the factor team performance also influenced the factor outcome (0.16). The paths from crowd size to the factor team activity and that from the factor referee's decisions to the factor outcome were significant but the coefficients were negligible.

**Figure 5:**
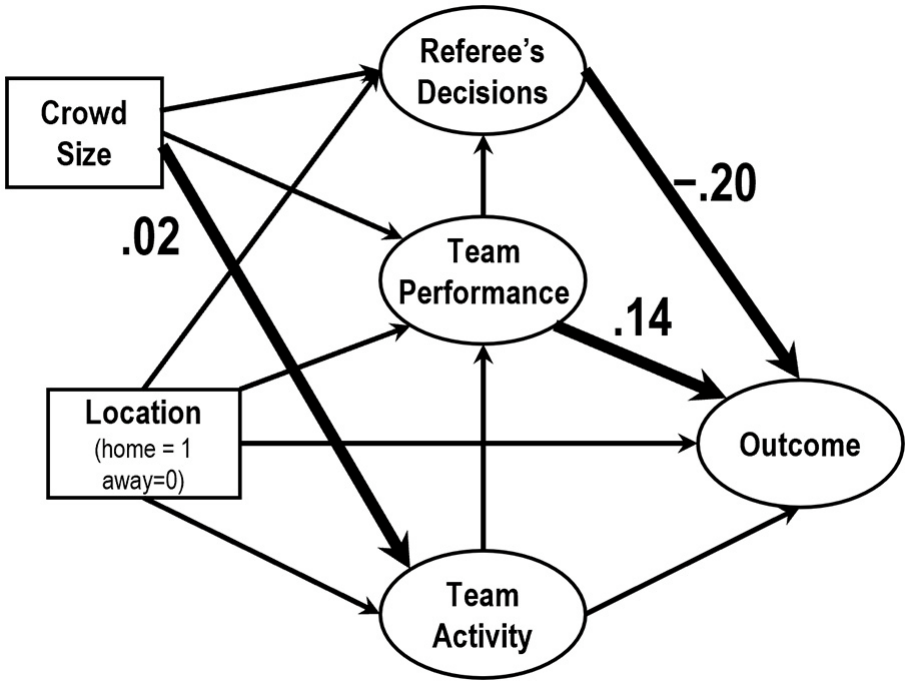
The results of SEM of data observed during 2020 season. The numbers show the statistically significant (*p* < 0.05) normalized coefficients. Non-significant coefficients were omitted.

Figure 5 depicts the SEM using matches played in 2020. The factor referee's decisions significantly influenced the factor outcome (0.20); the factor team performance influenced the factor outcome (0.14). The path from crowd size to the factor team activity was significant but the coefficients was negligible.

## Discussion

### Home advantage

In terms of points scored, HA was seen in 2019, which was a regular season; however, the difference between the mean point of home matches and that of the away matches was not significant in the 2020 season. This result suggests that HA in the J1 League disappeared during the period of COVID-19, as similar to the observations in many European Leagues (Almeida and Leite, 2021; Hill and Van Yperen, 2021; Leitner and Richlan, 2021; McCarrick et al., 2021). Because the crowd sizes were limited to a maximum of 20,000 people in the 2020 season, one possible interpretation of this result is that the influence of crowd size had diminished due to the restrictions in admission. Contrary to this, team activity consistently increased in the 2020 season than the 2019 season, regardless of home or away games. Small crowds had an effect of facilitating the team activity, although it remains unknown whether the effect due to imaging fans outside the stadium or not.

As for variables of factor team performance, the number of goal shots and corner kicks was consistently higher at home than away, regardless of the season. Although both variables had the potential to contribute to HA, they could not be explained by crowd size as they were attributed to the game location in both seasons. Thus, the effect of game location is even more influential while small crowds did increase the team activity. It is possible to deduce that the learning/familiarity and travel factors, as noted by Courneya and Carron (1992), are manifested in this effect. The characteristics of each stadium were not used as targets of analysis in my study because of which the factor that had the strongest influence on HA was not ascertained. Nonetheless, there is a need to identify this with more detailed studies in the future. With variables related to referee decisions, the number of warnings issued was consistently higher away than at home game. Thus far, this has been regarded as a variable liable to be influenced by crowd size. However, this too cannot be explained simply by crowd size as it was confirmed as the main effect of game location in both seasons. The number of send-offs had a low frequency, and its interaction was statistically negligible. The influence of crowd size on referees such as this has traditionally been regarded as a problem of psychological pressure (Leitner and Richlan, 2021; Wunderlich et al., 2021). Notably, these effects were also observed in 2020 as the matches played during that season had over 2,000 spectators except for the round 2 and 3 in the 2020 season. This suggests the possibility that although the crowd may have occupied a small percentage inside the stadium, they will have sufficient influence if they exceed a set number. This finding suggests that crowds in units of several thousands are sufficient to influence the referees, which supports the observations of researchers who stated that HA did not occur in amateur leagues that generally have small crowd sizes in the first place (Fischer and Haucap, 2021; Wunderlich et al., 2021).

The interpretations on three first step factors provide a perspective that increase of team activity would not simply lead team performance and/or referee's decisions to approach points won in the matches. Therefore, the process of HA would be considered.

### Routes through which crowd size influences the outcome

The results of a multi group SEM indicated that, at least in the J1 League, the crowd size weakly influenced referee's decision during 2019 seasons and the indirect effect from crowd size to outcome factor had almost no influence [0.13 × (−0.03) = 0.039]. This effect was no longer observed in the 2020 season when the number of spectators was restricted. In the 2020 season, however, crowd size did not significantly influence referee's decisions. In this season as well, the indirect effect from crowd size to outcome factor also had almost no influence. Thus, routes that crowd size influences the outcome was not found in the present study. The consistent influence found in this study was that from the team performance to the outcome, but this would be trivial from the viewpoint of football games.

## Conclusion

This study aimed to investigate the influence of crowd size on HA. The differences of points averagely won per game between home game and away game disappeared in J1 League in small crowd; and the team activity, i.e., the running distance and the number of sprits, increased during COVID-19. However, the influence of crowd size was weak and thus no dominant route was detected in the present study.

## Data availability statement

Publicly available datasets were analyzed in this study. This data can be found here: https://www.jleague.jp/stats/.

## Author contributions

RN conceived the study, analyzed the data, and wrote the manuscript.

## Funding

This study was supported by Japan Society for the Promotion of Science Grants-in-Aid for Scientific Research (C) (Grant No: JP21K12093).

## Conflict of interest

The author declares that the research was conducted in the absence of any commercial or financial relationships that could be construed as a potential conflict of interest.

## Publisher's note

All claims expressed in this article are solely those of the authors and do not necessarily represent those of their affiliated organizations, or those of the publisher, the editors and the reviewers. Any product that may be evaluated in this article, or claim that may be made by its manufacturer, is not guaranteed or endorsed by the publisher.
